# Cross-platform comparison of diabetic foot ulcer transcriptomes identifies partially concordant disease-associated signals

**DOI:** 10.1371/journal.pone.0356328

**Published:** 2026-10-27

**Authors:** Shiqin Huang, Yukun Li

**Affiliations:** 1 Department of Endocrinology, Zhangjiajie People’s Hospital, Zhangjiajie, Hunan, China; 2 Department of Gastroenterology, Shenzhen Xinhua Hospital, Shenzhen, Guangdong, China; Versiti Blood Research Institute, UNITED STATES OF AMERICA

## Abstract

The extent to which diabetic foot ulcer transcriptomic findings agree across cohorts remains uncertain. We compared an RNA sequencing cohort of 13 ulcer and eight diabetic foot skin samples with a microarray cohort of six ulcer and three diabetic foot skin samples. Single-cell data from 26 participants were analyzed after aggregating gene counts within each patient and cell type. A further cohort of 10 ulcer and 11 healthy foot-skin samples provided a descriptive directional comparison. Healing-status comparisons were exploratory. The primary analysis selected 4,704 differentially expressed gene identifiers; 2,943 were represented among the 17,502 gene symbols shared with the microarray cohort. Of these, 1,254 had concordant directions and nominal microarray P < 0.05, including 1,028 with microarray false discovery rate < 0.05. Direction agreement was 70.2%, and overall effect-rank correlation was modest (Spearman ρ = 0.29). Five Hallmark gene sets met the cross-cohort enrichment criteria. More specific functional analysis of the stricter supported set highlighted cornified-envelope formation, keratinization and inflammatory-cell migration. In the additional cohort, 611 of 823 represented genes from this set agreed in direction, although its comparator differed. Twelve keratinocyte genes met the cell-type differential-expression threshold; this finding does not establish cell-type exclusivity. Healing-status results were sensitive to covariate adjustment. These data support partial concordance in ulcer-associated epithelial, inflammatory and metabolic expression patterns. Limited cohort coverage, comparator differences and unresolved confounding preclude a universal disease signature, healing predictor or causal interpretation.

## Introduction

Diabetic foot ulcers (DFUs) are a severe complication of diabetes and a major proximal cause of infection, lower-extremity amputation, recurrent hospitalization and death [1–3]. Their clinical course reflects interacting neuropathic, vascular, mechanical, microbial and inflammatory processes, together with impaired re-epithelialization and extracellular-matrix remodeling [1–3]. This biological heterogeneity also makes it necessary to distinguish molecular changes associated with an established ulcer from those associated with subsequent healing.

Transcriptomic studies have identified numerous DFU-associated genes and pathways, but the reproducibility of these findings across independent cohorts and assay platforms remains uncertain. Small sample sizes, differences in tissue sampling and clinical characteristics, and platform-specific measurement may affect both the direction and magnitude of estimated expression differences. Identifying signals that remain directionally consistent across independent datasets is important before individual genes or pathways are prioritized for mechanistic or translational investigation.

Several public datasets provide complementary but non-equivalent views of DFU biology. GSE134431 profiled DFU tissue and diabetic foot skin (DFS) [4], GSE80178 examined a separate series of DFU and foot-skin samples using microarrays [5], and GSE165816 characterized foot, forearm and blood specimens at single-cell resolution [6]. These resources have supported subsequent studies of healing-associated genes, cellular states and potential therapeutic targets [7–9]. However, an established-ulcer contrast cannot by itself support prognosis, and reanalysis of the same participants does not provide independent cohort evidence.

We therefore evaluated the cross-cohort concordance of DFU-versus-DFS expression differences and used patient-level single-cell data to examine the broad cellular context of supported genes. We also explored the sensitivity of nonhealer-versus-healer findings to model specification. The primary objective was to identify disease-state associations with support across independent cohorts; the healing-status analyses were exploratory.

## Materials and methods

### Study design and public data sources

Analyses proceeded from the primary bulk RNA sequencing cohort to cross-cohort and cell-type evaluation (Fig 1; Table 1). We first processed the raw reads from GSE134431/SRP215049 and estimated DFU-versus-DFS differential expression in 13 DFU and eight DFS libraries. We then assessed gene- and pathway-level concordance in GSE80178, which contained six DFU, three DFS and three nondiabetic foot-skin arrays. Genes supported by both bulk cohorts were functionally annotated and compared descriptively with GSE199939, comprising 10 DFU and 11 healthy foot-skin samples. Finally, GSE165816 was analyzed using participant-level profiles to examine disease-associated expression within broad cell types. Nonhealer-versus-healer comparisons were exploratory. All data were obtained from the National Center for Biotechnology Information Gene Expression Omnibus (GEO).

**Table 1:** Cohort groups and roles in the analysis.

Accession	Assay	Group	n	Role
GSE134431	Bulk RNA sequencing	DFS	8	DFU versus DFS
GSE134431	Bulk RNA sequencing	DFU: healer/ nonhealer	7/ 6	Disease state; exploratory healing
GSE80178	Microarray	DFS/ DFU	3/ 6	DFU versus DFS
GSE80178	Microarray	Nondiabetic foot skin	3	Normalization; secondary comparison
GSE165816	Single-cell RNA sequencing	Diabetes without DFU	6	DFU versus diabetes
GSE165816	Single-cell RNA sequencing	DFU: healer/ nonhealer	7/ 4	Disease state; exploratory healing
GSE165816	Single-cell RNA sequencing	Without diabetes	9	Descriptive context
GSE199939	Bulk RNA sequencing; supplied TPM	DFU/ healthy foot skin	10/ 11	Descriptive direction only

For the bulk cohorts, n is the number of analyzed libraries/arrays; for GSE165816, n is the number of independent participants. Repeated foot matrices were aggregated per participant. DFS, diabetic foot skin; DFU, diabetic foot ulcer; TPM, transcripts per million. GSE199939 has a healthy comparator and is analyzed descriptively.

**Fig 1:**
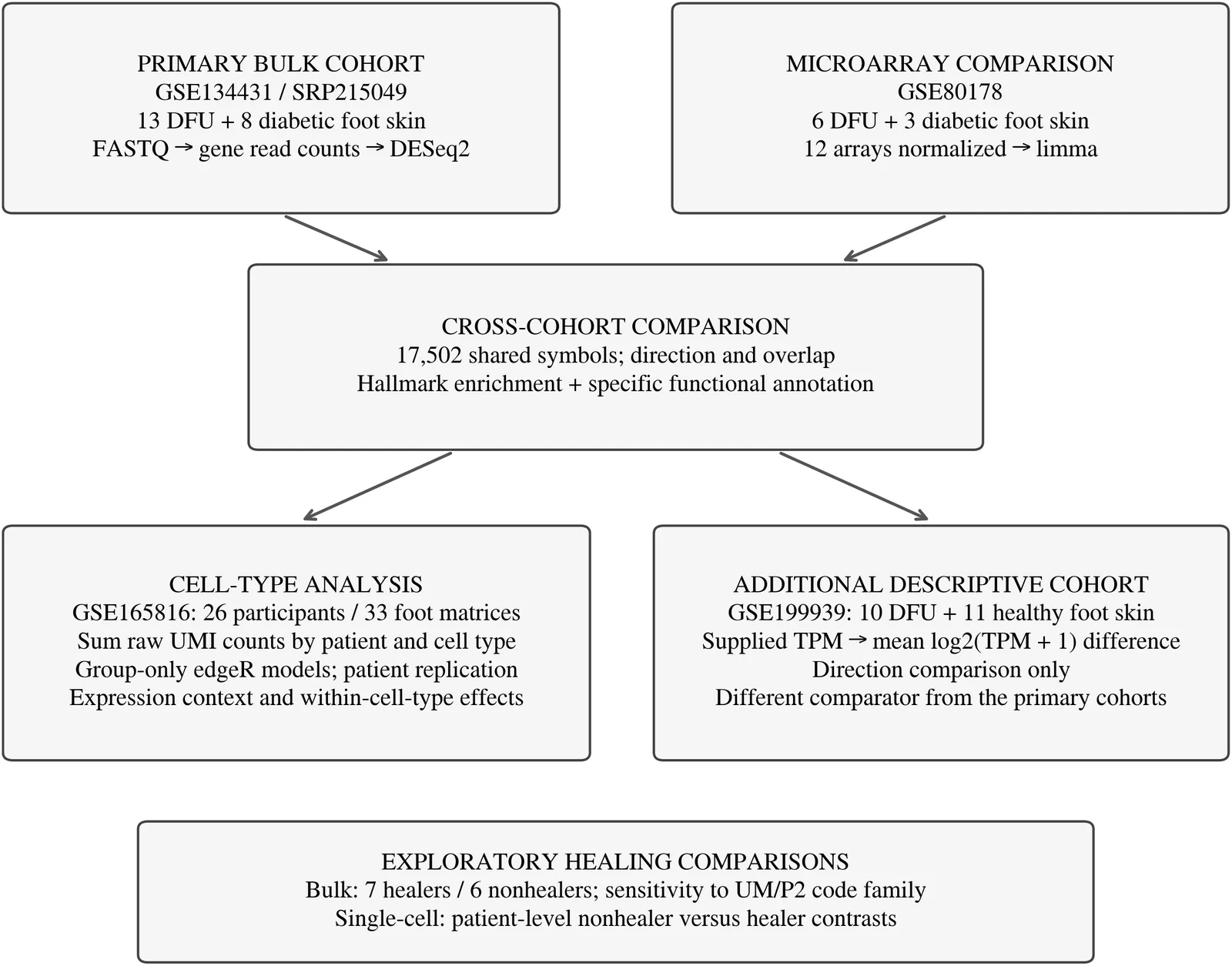
Sequential workflow for cross-platform and cell-type analyses. GSE134431/SRP215049 provided the primary RNA sequencing comparison of DFU with DFS. GSE80178 was used to assess cross-cohort gene and pathway concordance. Supported genes were functionally annotated and compared descriptively with GSE199939, which used healthy foot skin as the comparator. GSE165816 was analyzed using participant-level profiles within broad cell types. Nonhealer-versus-healer analyses were exploratory.

GSE165816 provided 54 specimen-level count matrices from foot skin, forearm skin and peripheral blood [6]. Specimen-to-participant relationships were determined from the publicly available participant metadata of the source study. The 33 foot matrices used here represented 26 participants: nine without diabetes, six with diabetes but no DFU, seven DFU healers and four DFU nonhealers. Repeated foot specimens were combined within participant before differential-expression testing. Thus, specimen counts and cell counts were not treated as numbers of independent participants.

A targeted GEO search conducted on 6 September 2026 returned 39 series records for (“diabetic foot” OR “diabetic ulcer” OR “diabetic ulcers”) AND Homo sapiens, restricted to series entries. We reviewed tissue, comparator, assay and independence information. Direct disease-state comparisons required patient-derived foot tissue with an ulcer and a diabetic nonulcerated comparator. Cultured-cell perturbations, non-foot tissues, different clinical contrasts and duplicate series were not pooled with this comparison. GSE199939 was retained separately because its comparator was healthy foot skin [10]. The screening decisions and source records are provided in S1 Text and S2 File.

### Primary RNA sequencing data processing

The GRCh38 primary-assembly FASTA and GENCODE Human release 50 primary-assembly GTF were obtained from the official GENCODE repository and verified against the release-level MD5 manifest [11]. Each FASTQ file was downloaded from the European Nucleotide Archive and accepted only when both its byte count and MD5 checksum matched the run manifest.

Read quality was assessed using FastQC v0.12.1 (https://www.bioinformatics.babraham.ac.uk/projects/fastqc/) and summarized using MultiQC v1.35 [12]. Across 210 library-by-module assessments, 129 were PASS, 54 were WARN and 27 were FAIL. Adapter Content, Per base sequence quality, Per sequence quality scores, Per base N content and Sequence Length Distribution were PASS in all 21 libraries. The FAIL calls concerned Per base sequence content (1), Per sequence GC content (11) and Sequence Duplication Levels (15); Overrepresented sequences generated 19 WARN and no FAIL calls. No trimming was applied because the adapter and base-quality modules passed in every library. S1 Fig summarizes read quality; the complete MultiQC report and per-library module table are supplied as S1 File and S1 Table.

Reads were aligned to GRCh38 using Rsubread v2.26.0 [13]. featureCounts generated gene counts from GENCODE exons grouped by gene_id; secondary alignments, multimapping reads and reads with mapping quality <10 were excluded. Alignment allowed no more than three mismatches and five inserted or deleted bases. Because the source study described directional library preparation, counts were generated in unstranded, forward-stranded and reverse-stranded modes for every library. The orientation with the greater phenotype-blind mean assigned-read count was selected before outcome modeling. The resulting matrix contained unnormalized integers.

### Primary RNA sequencing differential expression and pathway analysis

Genes with at least 10 counts in at least three samples were retained. DESeq2 v1.52.0 was used with a design containing disease condition and DFS as the reference level [14]. Wald tests estimated DFU-versus-DFS differences, and the Benjamini-Hochberg procedure controlled the false discovery rate (FDR) [15]. Normal-prior shrinkage was applied to log2 fold changes for ranking, visualization and the fold-change criterion. The primary gene-selection criteria were FDR < 0.05 and absolute shrunken log2 fold change ≥ 1. The FDR and fold-change criteria were applied jointly; unfiltered results were retained.

Variance-stabilized counts were used for principal component analysis (PCA) and the sample heatmap, but not for differential-expression testing. Ranked Hallmark enrichment used the signed DESeq2 Wald statistic, the Molecular Signatures Database Hallmark collection obtained using msigdbr v26.1.0 [16] and the multilevel fgsea v1.38.0 algorithm. FDR < 0.05 defined primary-cohort enrichment.

The nonhealer-versus-healer comparison used the 13 DFU samples (Table 2). An unadjusted DESeq2 model included healing group alone, and a sensitivity model also included UM/P2 sample-code family. We compared effect estimates, direction agreement, overlap and Jaccard similarity between sets with FDR < 0.10, together with design-matrix properties and sample-level Cook’s distances. This analysis was considered exploratory because the groups were small, sample-code family was imbalanced and clinically relevant prognostic covariates were not adequately captured. UM and P2 were treated as recorded sample-code categories rather than a fully characterized technical batch. In the separate DFU-versus-DFS analysis, sample-code family and disease condition were structurally confounded and could not be estimated simultaneously.

**Table 2:** Healing group and sample-code family in GSE134431.

Healing group	UM	P2	Total
Healer	6	1	7
Nonhealer	3	3	6
Total	9	4	13

The 13 DFU samples form the exploratory healing comparison. Both code families occur in both groups, so healing and UM/P2 are imbalanced but not perfectly confounded. Individual identifiers and original W0/W4 title labels are in Table B of S1 Text. Clinical covariates were insufficient for a prognostic model. Sample-code family is structurally confounded with condition in the separate DFU-versus-DFS analysis.

### Microarray analysis and cross-cohort concordance

All 12 GSE80178 CEL files were read with oligo v1.76.0 and jointly normalized using robust multi-array average (RMA) [17]. Transcript-cluster identifiers were mapped to gene symbols with the Bioconductor Human Gene 2.0 ST annotation package. Multiple expression summaries assigned to the same gene symbol were averaged. The main limma v3.68.4 contrast compared the six DFU and three DFS samples, using empirical Bayes variance moderation with robust estimation and a mean-variance trend [18]. DFS versus nondiabetic foot skin was a secondary contrast.

One DFU array, GSM2114232, separated from the other DFU arrays in principal component analysis and showed the greatest relative log-expression spread (S3 Fig). The analysis retaining all arrays remained primary. A post hoc sensitivity analysis excluded GSM2114232 and compared gene-effect estimates, direction agreement and retention of the primary FDR-positive gene set.

Cross-cohort concordance was evaluated among the 17,502 gene symbols shared by the two bulk datasets. For primary-selected genes represented in this shared set, we calculated direction agreement and reported microarray support at nominal P < 0.05 and FDR < 0.05; concordant effect direction was required for both tiers. A Venn diagram compared the primary-selected set with microarray FDR-positive genes, and the intersection was separated into concordant and opposite directions. The primary microarray set had no fold-change threshold; sensitivity summaries added absolute log2 fold-change thresholds of 0.5 and 1. Spearman correlation was used only as a descriptive summary of effect ranks. Hallmark gene sets met the cross-cohort criterion when primary FDR was < 0.05, microarray FDR was < 0.10 and normalized enrichment-score (NES) directions agreed.

### Functional annotation and descriptive comparison with an additional cohort

We performed overrepresentation analysis of the 1,028 genes with concordant directions and FDR < 0.05 in both bulk cohorts, retaining the primary fold-change criterion. The 561 upregulated and 467 downregulated genes were tested separately against Reactome Pathways 2024 and Gene Ontology Biological Process 2026 collections distributed by Enrichr [19–21] and downloaded on 6 September 2026. All 17,502 shared symbols formed the background. Terms containing 15–500 background genes were evaluated using an upper-tail hypergeometric test, and Benjamini-Hochberg adjustment covered all 7,764 tests across both collections and directions. This analysis provided more specific annotation of the cross-cohort-supported gene set.

GSE199939 provides transcripts-per-million (TPM) values rather than integer counts. TPM values for duplicated symbols were summed, and symbols with TPM ≥ 1 in at least three samples were retained. For each gene, we calculated the difference in mean log2(TPM + 1) between 10 DFU and 11 healthy samples and compared its direction with the primary supported set. Because the available data were TPM values and the comparator differed from the main contrast, this cohort was used only for descriptive directional comparison.

### Single-cell processing and patient-level differential expression

GSE165816 foot matrices contained raw unique molecular identifier counts with genes in rows and cell barcodes in columns. Cells expressing <200 genes or with >50% mitochondrial counts were excluded, and genes detected in <3 cells were removed, matching the source study’s documented filters [6]. Counts were normalized to 10,000 per cell and log transformed only for dimension reduction and annotation; raw counts were preserved separately.

Three thousand highly variable genes were selected using specimen as the batch stratum. A 50-component principal component representation was used to construct a graph with 20 neighbors, followed by uniform manifold approximation and projection (UMAP) and Leiden clustering with a fixed random seed. Broad cell types were assigned using cluster-average expression and marker genes (S5 Fig). Cluster markers and annotation scores were retained. This annotation was intended to identify broad lineages and did not resolve all cellular states described in the source study.

Raw unique molecular identifier (UMI) counts were summed within each participant and broad cell type to create pseudobulk profiles. Repeated foot specimens contributed to one profile per participant and cell type. Profiles required at least 50 cells and 10,000 total UMIs, and a contrast required at least three eligible participants per group. Within each eligible cell type, edgeR v4.10.1 applied filterByExpr with minimum count 10, trimmed mean of M-values normalization, robust dispersion estimation and quasi-likelihood testing [22]. Models contained clinical group alone; the participant was the unit of replication [23,24]. DFU healers and nonhealers were combined for comparison with diabetic participants without DFU. Nonhealer versus healer was a separate exploratory contrast. Gene-level criteria were FDR < 0.05 and absolute log2 fold change ≥ 1. Hallmark analysis used genes ranked by the signed square root of the quasi-likelihood F statistic.

### Cross-dataset integration and software

Gene symbols were used to link the bulk and single-cell results. RNA sequencing identifiers without a symbol were excluded; when multiple identifiers mapped to the same symbol, the entry with the smallest adjusted P value was retained, consistent with the archived integration script. Single-cell analyses reported expression abundance separately from within-cell-type differential expression.

The main analyses used R v4.6.1, Bioconductor v3.23, Python v3.13.13 and Scanpy v1.11.5 [25], as recorded in the archived sessions. Supplementary analyses used Python v3.12.13, NumPy v2.3.5, pandas v2.2.3 and SciPy v1.17.0. Code, inputs, complete results and execution instructions are supplied in S2 File.

### Ethics and data availability

This secondary analysis used de-identified public data and involved no new recruitment, intervention or specimen collection. Ethical approvals and consent procedures are described in the source studies [4–6,10]. Public source records are available under GEO accessions GSE134431, GSE80178, GSE165816, GSE199939 and GSE166120 and Sequence Read Archive accession SRP215049. GSE165816 provides processed single-cell count matrices; its raw sequencing reads are withheld by the data generators for participant privacy. The original analysis code, count matrices and complete results are openly archived at https://doi.org/10.5281/zenodo.21740724. S1 Table retains the original tabulations; S2 File supplies the additional code, frozen inputs, cohort-screening records and complete revision-analysis results.

### Use of artificial intelligence-assisted tools

ChatGPT (OpenAI) was used as an assistive tool for language editing and document preparation and to help draft and check supplementary analysis and plotting scripts. The authors defined the research question, made all analytical and interpretive decisions, verified numerical outputs against the public data and archived results, and checked citations against the original publications. The authors reviewed the final manuscript and supporting files and accept full responsibility for their content.

## Results

### Sequencing quality and gene-level counts

All 21 single-end FASTQ files matched the European Nucleotide Archive byte counts and MD5 checksums. The libraries contained 1.18 billion reads in total (median, 49.5 million; range, 32.4–103.5 million). Median mapping was 95.6% (range, 89.6–98.2%), with a median of 83.3% uniquely mapped reads. The complete FastQC status distribution was 129 PASS, 54 WARN and 27 FAIL calls (S1 Fig). None of the FAIL calls concerned adapter content or base-quality modules, and no trimming was applied. Reverse-stranded counting produced the greater phenotype-blind mean assignment and was selected. In total, 613.0 million reads were assigned to genes (median, 27.4 million per library), yielding an unnormalized integer count matrix.

Quality-control (QC) summaries stratified by disease condition and sample-code family are provided in S1 Table. Median total reads and mapping rates were 52.59 million and 95.15% for DFS donor-code libraries (n = 8), 50.94 million and 95.60% for DFU P2 libraries (n = 4), and 45.58 million and 96.79% for DFU UM libraries (n = 9). PCA was annotated by disease condition and sample-code family (Fig 2). Because DFS and DFU samples occupy different provenance-code structures, these diagnostics cannot establish that every observed condition effect is biological.

**Fig 2:**
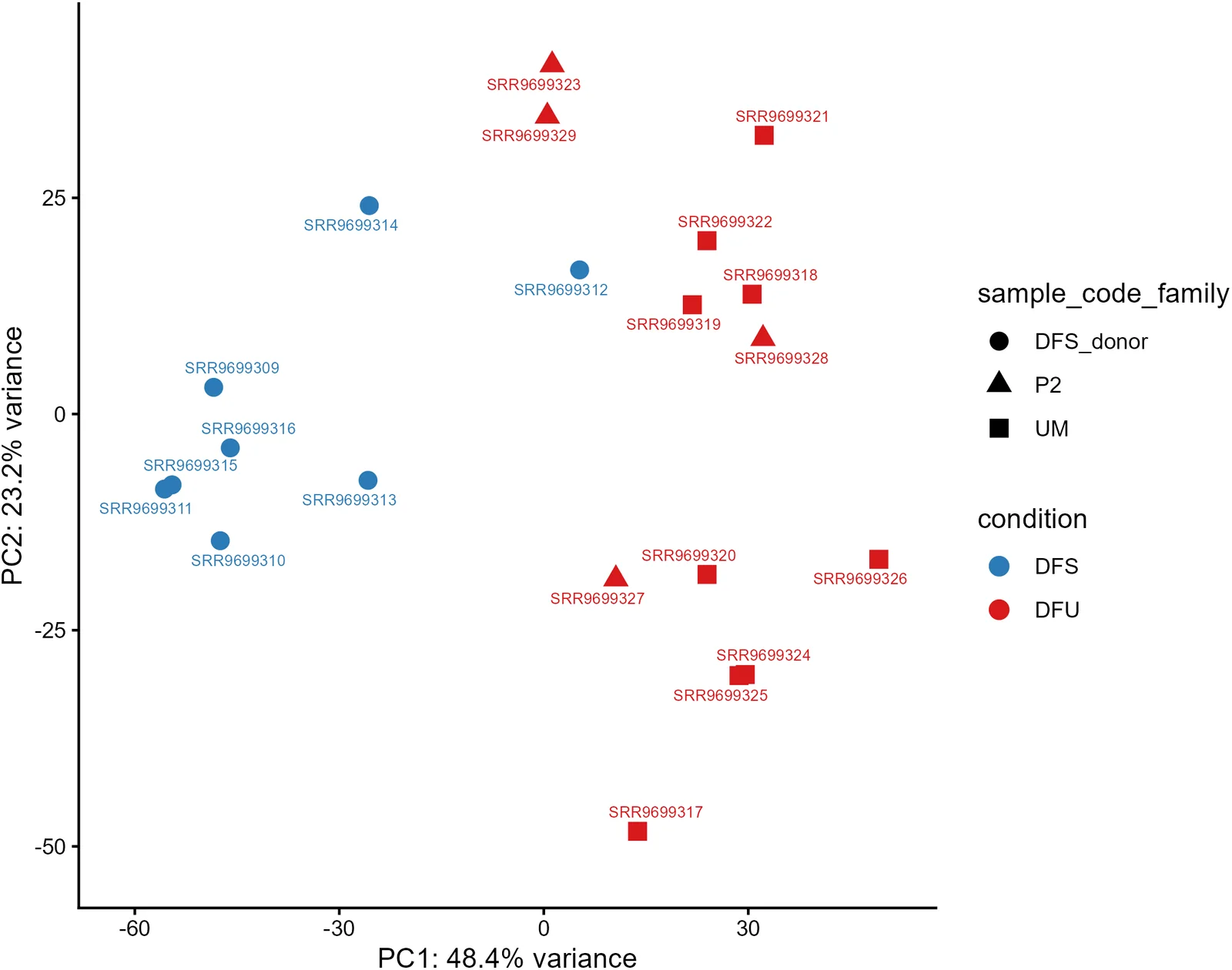
Sample structure of the primary RNA sequencing cohort. Principal component analysis of variance-stabilized GSE134431 counts. Color indicates DFU or DFS, and shape indicates sample-code family. Disease condition and sample provenance cannot be separated in this cohort.

### Primary RNA sequencing identifies widespread DFU-associated expression differences

DESeq2 tested 26,717 gene identifiers, of which 4,704 met FDR < 0.05 and absolute shrunken log2 fold change ≥ 1: 1,869 had higher and 2,835 had lower expression in DFU. These are within-cohort associations. Thirty-seven Hallmark gene sets met FDR < 0.05. Positively enriched sets included mechanistic target of rapamycin complex 1 signaling, tumor necrosis factor-alpha signaling via nuclear factor-kappa B, MYC targets v1, protein secretion and hypoxia. Interferon-alpha response and KRAS signaling down were negatively enriched (Figs 3 and 4; S2 Fig). The latter is a gene-set name and does not directly measure KRAS activity.

**Fig 3:**
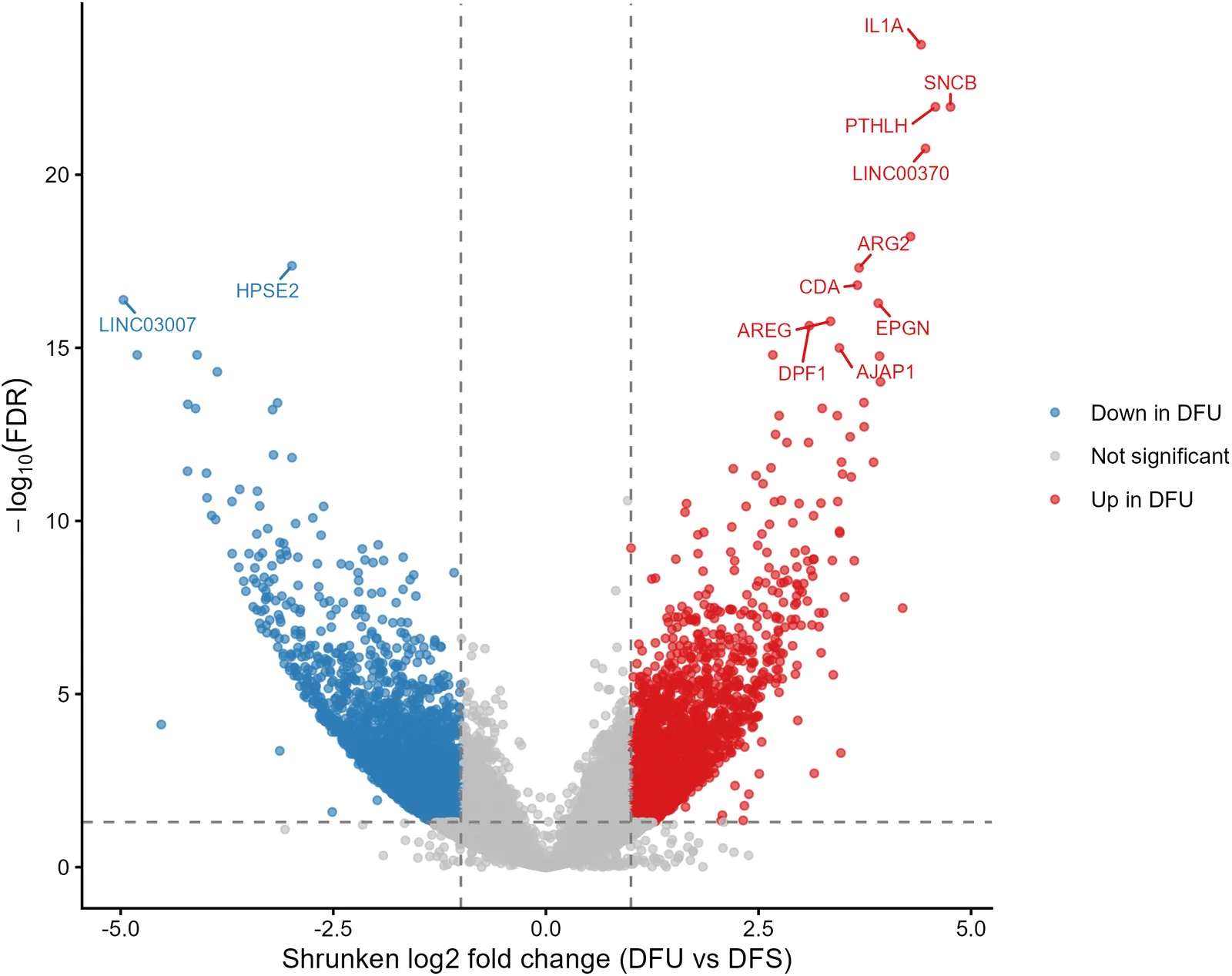
DFU-associated differential expression in the primary RNA sequencing cohort. The volcano plot shows shrunken log2 fold changes from DESeq2 for DFU versus DFS. Highlighted genes meet FDR < 0.05 and absolute shrunken log2 fold change ≥ 1.

**Fig 4:**
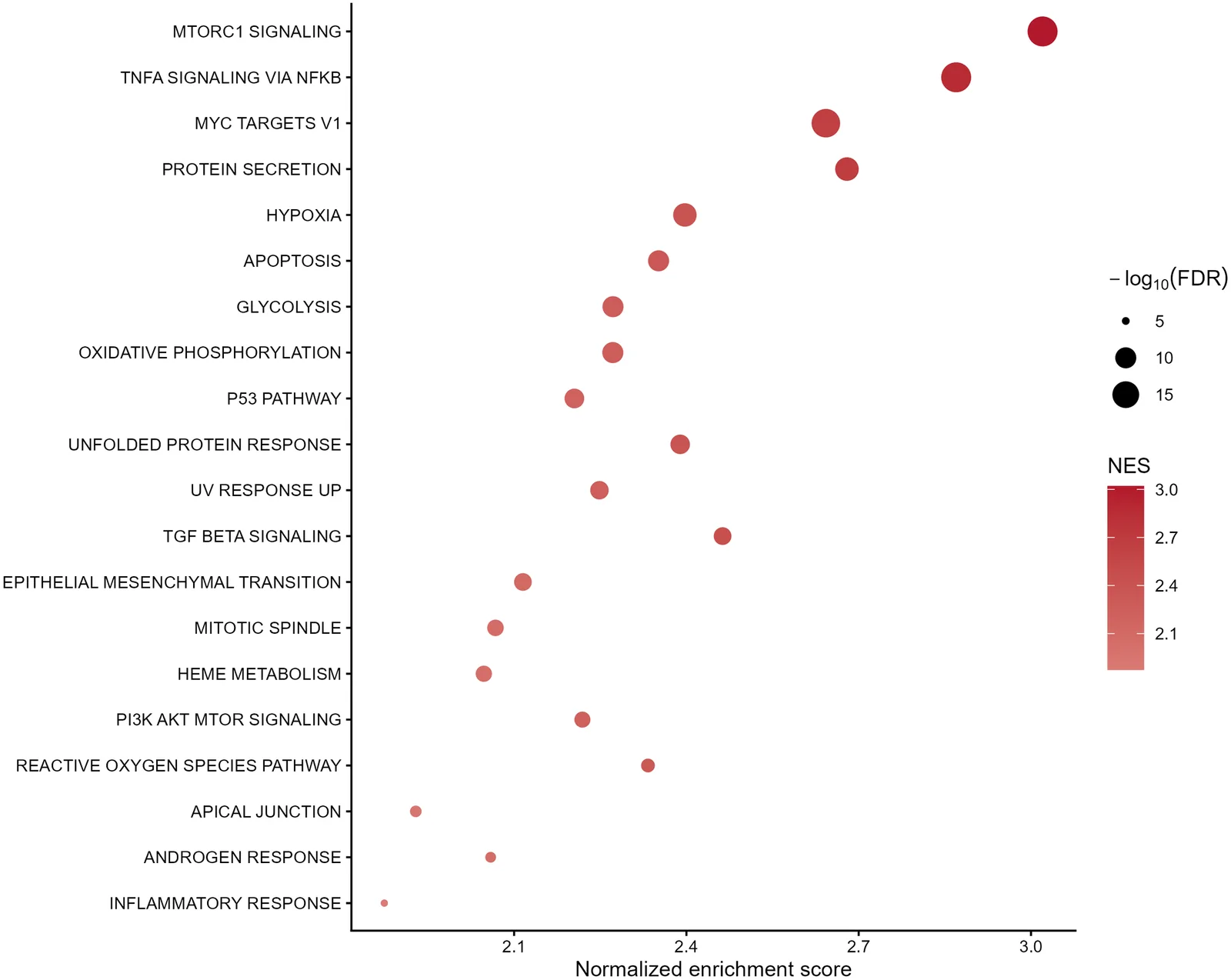
Hallmark pathway enrichment in the primary RNA sequencing cohort. Normalized enrichment scores were calculated by fgsea using genes ranked by the signed DESeq2 Wald statistic. Point size indicates -log10(FDR). More specific Reactome and Gene Ontology annotations are shown in S7 Fig.

### The microarray cohort provides partial directional support

The microarray analysis tested 27,277 gene symbols, of which 17,502 were shared with the RNA sequencing results (Fig 5). Among the 4,704 primary-selected gene identifiers, 1,399 lacked a symbol. The remaining identifiers mapped to 3,303 unique symbols, including 2,943 represented in the shared set. Of these 2,943 genes, 2,065 (70.2%) agreed in direction, 1,254 had concordant effects with nominal microarray P < 0.05, and 1,028 also had microarray FDR < 0.05. Across all shared symbols, the descriptive effect-rank correlation was modest (Spearman ρ = 0.29).

**Fig 5:**
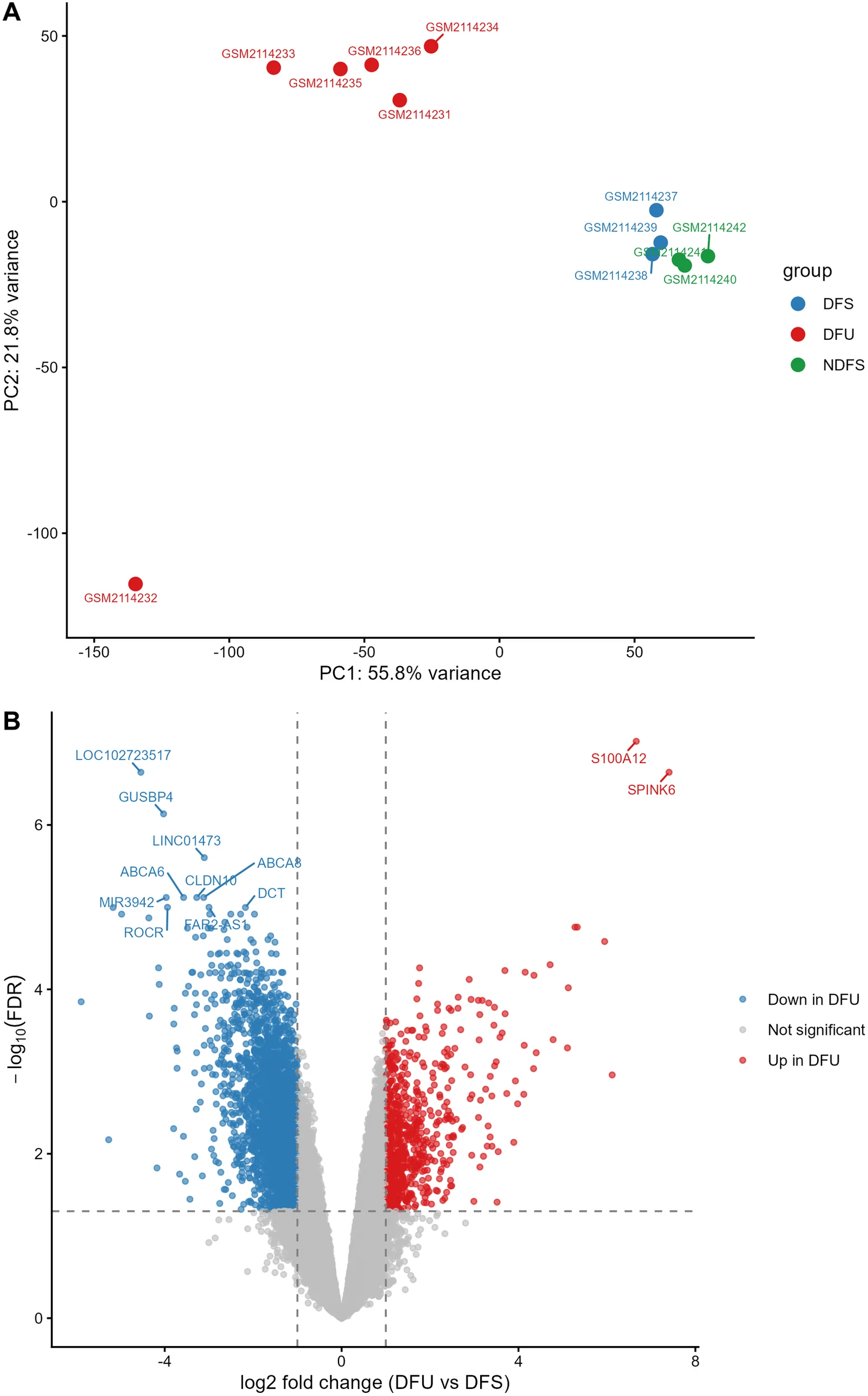
Sample structure and DFU-associated differential expression in the microarray cohort. (A) Principal component structure of RMA-normalized GSE80178 expression. (B) DFU-versus-DFS differential expression estimated with limma.

Within the shared universe, 2,943 genes met the primary selection criteria and 5,541 met microarray FDR < 0.05. The intersection contained 1,210 genes: 1,028 had concordant directions and 182 had opposite directions (Jaccard index, 0.166; Fig 6). Threshold sensitivity is provided in S2 File. Five Hallmark gene sets met the cross-cohort criterion: tumor necrosis factor-alpha signaling via nuclear factor-kappa B, hypoxia, glycolysis and inflammatory response were positively enriched, whereas interferon-alpha response was negatively enriched in both cohorts (Figs 6). Excluding GSM2114232 in the post hoc microarray sensitivity analysis produced similar effects (Spearman ρ = 0.956); 99.6% of the primary FDR-positive genes remained below FDR 0.05 and their directions were preserved (S4 Fig).

**Fig 6:**
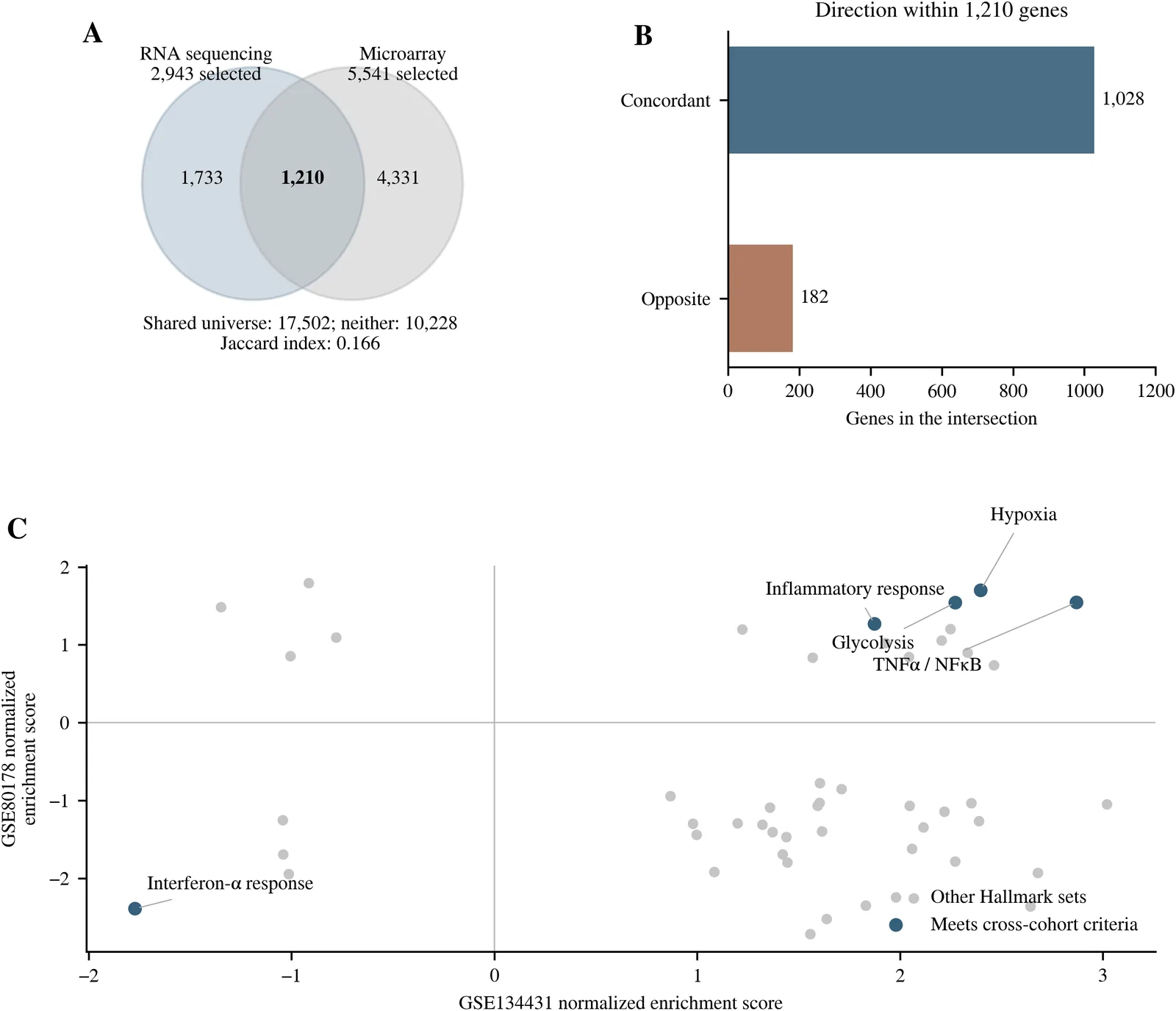
Partial cross-cohort concordance at the gene and pathway levels. (A) Overlap of significant gene sets among 17,502 shared symbols. The RNA sequencing set requires FDR < 0.05 and absolute shrunken log2 fold change ≥ 1; the microarray set requires FDR < 0.05. Counts are not area-scaled. (B) Effect direction within the 1,210-gene intersection: 1,028 genes are concordant and 182 are opposite. (C) Concordance of Hallmark normalized enrichment scores. Highlighted sets meet primary FDR < 0.05, microarray FDR < 0.10 and concordant directions.

### Specific functional annotation and descriptive evidence from GSE199939

Among 7,764 functional tests, 26 met global FDR < 0.05: 18 Gene Ontology terms and eight Reactome terms, all in the upregulated set. The strongest Reactome terms were formation of the cornified envelope (29 overlapping genes; FDR = 1.86 × 10^−14^) and keratinization (29 genes; FDR = 2.00 × 10^−14^). Gene Ontology results included keratinocyte differentiation (10 genes; FDR = 0.00152), epidermal cell differentiation (11 genes; FDR = 0.00173), granulocyte chemotaxis (10 genes; FDR = 0.0101) and regulation of wound healing (eight genes; FDR = 0.0246). No downregulated term met the global threshold (S7 Fig; S2 File). These results provide more specific biological annotation of the cross-cohort-supported genes.

The descriptive GSE199939 comparison retained 19,752 symbols, including 14,892 shared with the main bulk comparison. Of the stricter 1,028 supported genes, 823 were represented and 611 (74.2%) agreed in direction. Across all 14,892 shared symbols, the rank correlation with the primary effect was 0.37 (S8 Fig). This additional evidence concerns DFU versus healthy foot skin and cannot isolate ulcer effects from diabetes or other cohort differences.

### Patient-level analysis identifies keratinocyte-associated differences

The 33 foot matrices contained 94,325 filtered cells from 26 participants. Applying the stated quality thresholds removed no additional cells from these processed public matrices. Aggregation yielded 242 patient-by-cell-type profiles across 10 broad lineages; six had sufficient replication for comparison of DFU with diabetes without DFU (Table 3). Eligible patient numbers varied from three to six in the diabetic comparison group and from nine to 11 in the DFU group.

**Table 3:** Eligible patient replication for cell-type comparisons of DFU with diabetes without DFU.

Cell type	Diabetes without DFU	DFU	Genes tested	Genes meeting criteria
Endothelial	5	11	11,638	0
Fibroblast	6	11	12,617	0
Keratinocyte	5	10	12,193	12
Myeloid	3	9	9,117	0
Smooth muscle/ pericyte	5	11	12,779	0
T/ NK	5	11	5,715	0

Eligibility required ≥50 cells and ≥10,000 UMIs per patient-by-cell-type profile and ≥3 patients per group. Counts are independent patients, not individual cells. Genes meeting criteria have FDR < 0.05 and absolute log2 fold change ≥ 1. Other lineages did not meet the replication requirements. Different significance across lineages is not a test of a between-lineage difference.

Twelve keratinocyte genes met FDR < 0.05 and absolute log2 fold change ≥ 1: CARD6, SPRR2A, KYNU, SERPINB4, KCNJ15, S100A9, CLEC7A, C4orf48, C2CD4A, AKR1B10, SERPINB13 and S100A8. No gene met both criteria in the other eligible lineages, although this difference in statistical significance is not a test of a between-lineage difference. Keratinocyte Hallmark results included MYC targets v1, mechanistic target of rapamycin complex 1 signaling, interferon-gamma response, glycolysis, interferon-alpha response and inflammatory response. Other lineages showed pathway-level effects despite having no genes meeting the individual-gene threshold. Fig 7 shows the cell annotations, whereas Fig 8 summarizes expression abundance across broad cell types.

**Fig 7:**
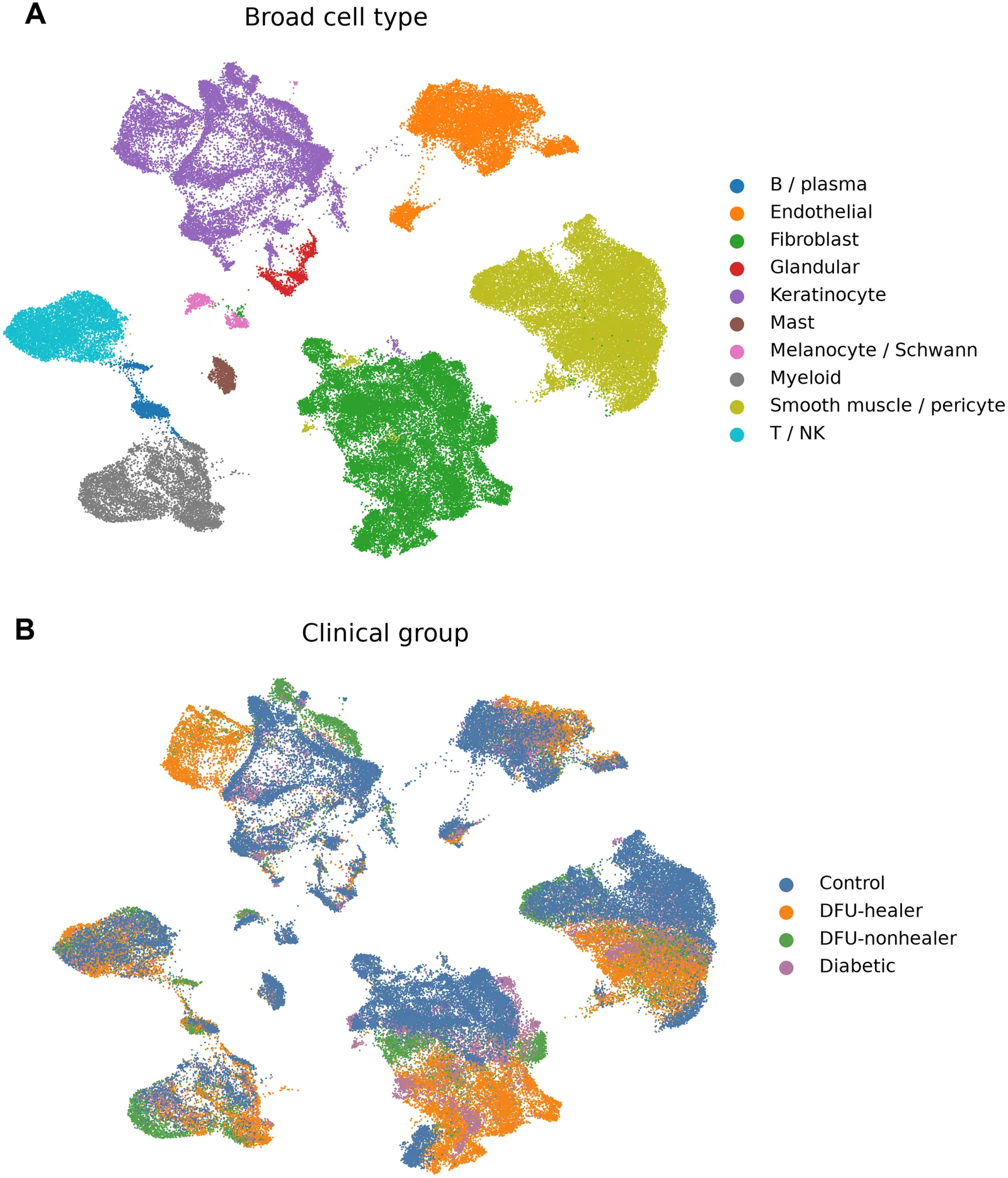
Broad cell types and clinical groups in the single-cell cohort. GSE165816 UMAP colored by (A) broad cell type and (B) clinical group. The embedding is descriptive; differential-expression testing used participant-level profiles.

**Fig 8:**
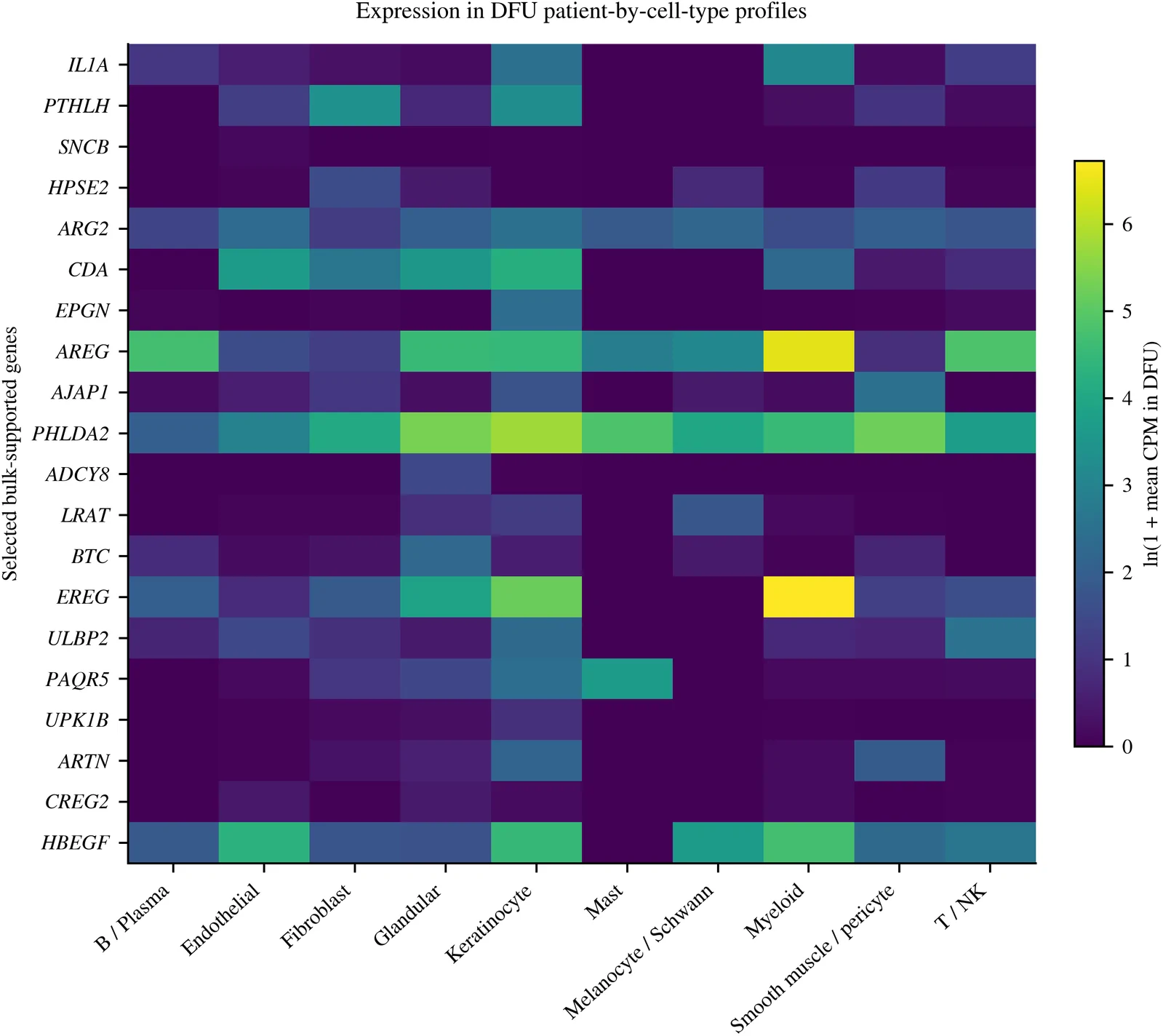
Broad cell-type expression of bulk-supported genes in DFU samples. The heatmap shows natural log(1 + mean counts per million) in eligible DFU participant-by-cell-type profiles. The 20 displayed genes are ordered by microarray FDR status, primary adjusted P value and microarray adjusted P value. The figure describes expression abundance and does not test differential expression between cell types or establish an exclusive cellular origin.

The source study’s spatial series GSE166120 was examined for coverage of the 12 keratinocyte genes. Its targeted panel included S100A8, S100A9 and CLEC7A, but not the other nine genes. The spatial experiment comprised four patients, two healers and two nonhealers, with multiple regions of interest per patient [6]. These data were not used to estimate a new spatial differential-expression result (S1 Text; S2 File).

### Healing-status results are sensitive to model specification

The unadjusted comparison of seven healers with six nonhealers produced 120 genes at FDR < 0.10, whereas the model including sample-code family produced 1,471. The models’ finite all-gene log2 fold changes were correlated (Spearman ρ = 0.755), and 78.8% agreed in direction. However, their FDR-positive sets shared only 111 genes, with nine unique to the unadjusted model and 1,360 unique to the adjusted model (Jaccard index, 0.075). Within the union of FDR-positive genes, effect sizes were strongly correlated (ρ = 0.950) and 99.7% agreed in direction. Thus, the difference between the two selected sets mainly reflected changes in estimated precision rather than widespread reversal of effects.

Sample-code family was imbalanced but not perfectly separated: healers comprised six UM and one P2 sample, whereas nonhealers comprised three UM and three P2 samples (Fisher’s exact P = 0.266). Cook’s-distance threshold exceedances occurred in several libraries rather than a single uniquely influential sample. No eligible single-cell lineage produced a healing-status gene meeting FDR < 0.05 and absolute log2 fold change ≥ 1. Given the marked sensitivity to model specification, neither bulk gene list was treated as definitive and neither supports a healing classifier (S6 Fig).

## Discussion

The primary bulk cohorts showed partial agreement in DFU-associated expression differences. Of 2,943 primary-selected symbols represented in both cohorts, 1,254 had concordant effects and nominal microarray evidence, and 1,028 also met microarray FDR < 0.05. Five Hallmark gene sets met the cross-cohort criteria. The modest all-gene correlation and incomplete gene-set overlap limit the strength of this evidence. More specific enrichment linked the stricter supported set to cornified-envelope formation, keratinization and inflammatory-cell migration. These findings describe epithelial and inflammatory components of the established-ulcer state, without establishing a universal signature.

The main contribution of this analysis is the separation of three related questions: disease-state association, support across independent cohorts and uncertainty in healing-status comparisons. Previous reanalyses of these resources have examined nonhealing-associated genes, fibroblast states and potential drug targets [7–9]. We used established count-based and patient-level pseudobulk methods [23,24,26–28] to quantify concordance rather than to introduce a new analytical method. Because we did not conduct matched benchmarking of every analytical choice, the present results complement that literature rather than correct it.

The keratinocyte findings are compatible with epithelial stress and altered differentiation, but statistical detection depends on participant numbers, cell yield, abundance and between-patient variability. Significance in one lineage and nonsignificance in another does not establish a difference between lineages. Broad annotation may also conceal subtypes or mixed profiles. The source study reported spatial associations involving fibroblasts and macrophages [6], and subsequent work investigated epidermal, stromal and immune changes [9,29–32]. Its GSE166120 spatial panel covered only three of the 12 keratinocyte genes identified here (*S100A8*, *S100A9* and *CLEC7A*; S1 Text). With two healers and two nonhealers and no DFS comparator, those targeted measurements cannot test the full disease-state candidate set. We therefore did not claim spatial confirmation or infer ligand-receptor mechanisms from expression alone. Refined annotation, adequately replicated spatial sampling and perturbation experiments remain necessary.

Healing-status comparisons were especially sensitive to model specification. Adjustment for UM/P2 sample-code family increased the number of genes below FDR 0.10 from 120 to 1,471, while directions were largely preserved among the selected genes. This pattern is consistent with changes in statistical precision and highlights the dependence of thresholded lists on a small, imbalanced sample. The nonsignificant Fisher test for the sample-code distribution does not establish covariate balance or rule out confounding. The absence of single-cell genes meeting the patient-level healing threshold is also not evidence that healing-associated biology is absent. Information on perfusion, infection, treatment, ulcer duration and off-loading was insufficient for a prognostic analysis.

Several limitations constrain interpretation. Sample-code family is structurally confounded with the primary DFU-versus-DFS contrast, and a second cohort cannot exclude shared clinical or sampling biases. The accession-based audit used an explicit GEO query but was not a systematic review. GSE199939 provided an additional directional comparison, but its healthy comparator does not separate diabetes from ulcer effects. Small groups, platform differences, heterogeneous tissue sampling and symbol mapping also restrict comparability; in particular, the archived integration script retained the smallest adjusted P value when multiple RNA sequencing identifiers mapped to one symbol. The single-cell analysis began with processed public matrices, used broad lineages and could not adjust comprehensively for clinical covariates. Finally, enrichment and expression concordance describe association rather than pathway activation, causal mechanism or predictive performance.

These public cohorts show partial concordance in ulcer-associated epithelial, inflammatory and metabolic expression patterns. Patient-level analyses identify keratinocyte-associated differences within a broader multicellular wound environment. Larger cohorts with harmonized sampling and clinical covariates are needed to assess the generalizability of these associations and their relationship to healing.

## Supporting information

S1 Fig GSE134431 sequencing quality summary. Per-library read counts and the complete FastQC PASS, WARN and FAIL distribution. The full interactive MultiQC report is supplied as S1 File.
(TIF)

S2 Fig Primary-cohort top-gene heatmap. Variance-stabilized expression of the 50 genes with the smallest adjusted P values in GSE134431.
(TIF)

S3 Fig GSE80178 array quality. Relative log-expression distributions after robust multi-array average normalization.
(TIF)

S4 Fig GSE80178 outlier sensitivity. Primary versus GSM2114232-excluded log2 fold changes. This was a labeled post hoc sensitivity analysis.
(TIF)

S5 Fig GSE165816 annotation evidence. Canonical-marker dot plot supporting the 10 broad cell classes.
(TIF)

S6 Fig Healing-model sensitivity. (A) Nonhealer-versus-healer log2 fold changes from unadjusted and sample-code-family-adjusted DESeq2 models. (B) Maximum Cook’s distance by sample under each model.
(TIF)

S7 Fig Specific functional annotation of concordant bulk genes. The five terms with the smallest global FDR in each collection are displayed. Dot size represents overlap count and the x-axis fold enrichment. Global adjustment includes both directions and both collections; all displayed terms are in the upregulated set. The complete 7,764 tests are in S2 File.
(TIF)

S8 Fig Descriptive comparison with GSE199939. (A) Direction agreement for the 823 represented genes from the stricter bulk-supported set. (B) Primary shrunken log2 fold change versus the difference in mean log2(TPM + 1) in GSE199939 across 14,892 shared symbols. The additional cohort compares DFU with healthy foot skin and is not a direct replication of DFU versus DFS.
(TIF)

S1 Table Complete metadata, quality-control metrics and statistical results from the original analysis. The workbook contains 31 worksheets covering cohort definitions, checksums, count matrices, DESeq2, limma and edgeR results, pathway analyses, patient mapping and cross-cohort comparisons.
(XLSX)

S1 Text Supplementary methods and results. Expanded acquisition and model details, cohort eligibility, patient grouping, cell-type replication, and supplementary revision analyses.
(DOCX)

S1 File Complete interactive MultiQC report. Standalone HTML report for all 21 GSE134431 FASTQ libraries.
(ZIP)

S2 File Supplementary revision analysis package. Executable scripts, frozen inputs, 39-series GEO screening log, complete gene-set overlap and enrichment results, GSE199939 descriptive comparisons, spatial-panel coverage audit, figure source data, checksums and reproduction instructions.
(ZIP)

## Abbreviations

DFS: diabetic foot skin
DFU: diabetic foot ulcer
FDR: false discovery rate
GEO: Gene Expression Omnibus
NES: normalized enrichment score
PCA: principal component analysis
RMA: robust multi-array average
TPM: transcripts per million
UMAP: uniform manifold approximation and projection
UMI: unique molecular identifier
FASTQ: the sequencing-read file format
CEL: the Affymetrix intensity-file format
FASTA: a sequence-file format
GTF: the gene-transfer annotation format
MD5 and SHA-256: file-integrity hash algorithms
