# Supplementary material for: Cross-platform comparison of diabetic foot ulcer transcriptomes identifies partially concordant disease-associated signals
Source: PLoS One. 2026 Oct 27;21(10):e0356328. doi: 10.1371/journal.pone.0356328 (PMC13712624; doi:10.1371/journal.pone.0356328)
Supplement: Supplementary file 1 [file pone.0356328.s010.docx]

S1 Text. Supplementary methods and results

Cross-platform comparison of diabetic foot ulcer transcriptomes identifies partially concordant disease-associated signals

Shiqin Huang and Yukun Li

# 1. Public data, acquisition and integrity

This supplement documents the original analysis and additional analyses performed during revision. The original public repository is https://doi.org/10.5281/zenodo.21740724 (version 1.0.0). S1 Table retains the original 31-sheet workbook; S2 File contains new scripts, frozen inputs, complete revision outputs and source records. The original R models were not refitted during this revision.

The 21 GSE134431/SRP215049 single-end FASTQ files were obtained from the European Nucleotide Archive endpoints in the run manifest. Byte counts and MD5 hashes were checked. GENCODE Human release 50 GRCh38 primary-assembly FASTA and GTF were checked against the official release manifest. The 12 GSE80178 CEL files and 54 GSE165816 specimen count matrices were downloaded from GEO. The public single-cell matrices contain UMI counts; the source investigators withhold the underlying raw sequencing reads for participant privacy.

# 2. Cohort audit during revision

On 6 September 2026, a targeted Entrez/GEO query returned 39 series records: ("diabetic foot"[All Fields] OR "diabetic ulcer"[All Fields] OR "diabetic ulcers"[All Fields]) AND Homo sapiens[Organism] AND gse[Entry Type]. We reviewed series-level tissue, comparator, assay and design information. A series entry or specimen was not counted as an independent patient, and related accessions were not assumed to be independent cohorts. The complete decisions and frozen series records are in S2 File.

The primary question required patient-derived foot tissue comparing DFU with nonulcerated diabetic foot skin. Studies of forearm skin, isolated cells, cell perturbations, blood or arterial tissue were not pooled with the primary contrast. Intervention and healing-only comparisons address different questions. GSE199939 was selected separately as a descriptive comparison with a healthy foot-skin comparator. This was a targeted audit, not a preregistered systematic review, and not every series with a different comparator was reanalyzed. For example, GSE286165 compares DFU with normal foot-trauma tissue and is listed transparently as not pooled.

Table A. Selected accessions illustrating the cohort decisions.

| **Accession** | **Use** | **Reason** |
| --- | --- | --- |
| GSE134431 | Main bulk analysis | Human DFU versus nonulcerated diabetic foot skin; 13 versus 8 libraries. |
| GSE80178 | Main bulk comparison | Human DFU versus diabetic foot skin; 6 versus 3 arrays; 3 additional healthy arrays used for normalization. |
| GSE165816 | Main single-cell analysis | Foot matrices and participant mapping support patient-level comparisons; 33 foot matrices represent 26 participants. |
| GSE199939 | Additional descriptive comparison | 10 DFU versus 11 healthy foot-skin samples. Different comparator; supplied TPM analyzed descriptively without differential-expression P values. |
| GSE143735 | Not pooled | Forearm skin in diabetic participants with or without foot ulceration; anatomically distinct from sampled foot wounds. |
| GSE223964 | Not pooled | Single-cell chronic diabetic versus nondiabetic wounds; lacks the matched nonulcerated diabetic comparator. |
| GSE245703 | Not pooled | Single-cell chronic diabetic versus nondiabetic wounds; different comparator. Independence from related wound series not assumed. |
| GSE231643 | Not pooled | Healing versus nonhealing DFU debridement single-cell profiles; no nonulcerated diabetic comparator. Relevant for future healing-focused analysis. |
| GSE166120 | Spatial panel coverage audit | Four patients (2 healers, 2 nonhealers); targeted panel and no DFS comparator. Panel coverage only, not a new spatial expression analysis. |
| GSE286165 | Not pooled | DFU versus normal foot-trauma tissue (3 per group), with circRNA-focused study aims. Different comparator; no direct DFU-versus-DFS estimate was added. Not all differently controlled cohorts were reanalyzed. |
| GSE219078 | Not pooled | Isolated human dermal microvascular endothelial cells, rather than intact foot-tissue profiles. |
| GSE230426 | Not pooled | Peripheral-blood transcriptomes before and after treatment of infection; different tissue and longitudinal contrast. |

All 39 records and source URLs are in S2 File. GEO sample totals describe deposited specimens or libraries and may differ from independent patient counts.

# 3. Primary RNA sequencing: quality control and quantification

FastQC v0.12.1 and MultiQC v1.35 summarized read quality. Across 21 libraries and 10 modules, 129 calls were PASS, 54 WARN and 27 FAIL. Adapter content, per-base quality, per-sequence quality, N content and sequence-length distribution passed in all libraries. Overrepresented sequences gave two PASS and 19 WARN calls. Per-base sequence content gave 20 WARN and one FAIL; GC content gave one PASS, nine WARN and 11 FAIL; duplication gave six WARN and 15 FAIL. Basic statistics also passed in all 21 libraries. S1 Fig, S1 File and S1 Table provide the detailed records.

No adapter or low-quality-tail trimming was performed because all corresponding modules passed. Composition, GC and duplication flags can reflect library complexity or biology and are not necessarily corrected by terminal trimming. The original analysis did not compare trimmed with untrimmed results. Rsubread v2.26.0 aligned reads to GRCh38; featureCounts used exons grouped by gene_id, mapping quality ≥10, and excluded secondary, multimapping and ambiguous multi-feature assignments. The original optional STAR script is an alternative route and did not generate the reported results. Among unstranded, forward and reverse counting, reverse-stranded counting gave the greatest phenotype-blind mean assignment and was selected.

All 21 FASTQ files matched their manifest. Libraries contained 1.18 billion reads (median 49.5 million; range 32.4–103.5 million). Median mapping was 95.6% (range 89.6–98.2%) and median unique mapping was 83.3%. Reverse counting assigned 613.0 million reads to genes (median 27.4 million per library). Median reads and mapping rates were 52.59 million/95.15% in eight DFS donor libraries, 50.94 million/95.60% in four DFU P2 libraries, and 45.58 million/96.79% in nine DFU UM libraries. These diagnostics do not resolve condition–provenance confounding. Fig 2 shows disease condition and sample-code family separately.

# 4. Primary and healing-status models

The primary DESeq2 model was ~ condition, with DFS as reference. Genes with ≥10 counts in ≥3 samples were retained. Wald-test P values were adjusted by Benjamini–Hochberg. Primary selection required FDR < 0.05 and absolute normally shrunken log2 fold change ≥1. Of 26,717 tested identifiers, 4,704 met both criteria (1,869 up; 2,835 down). All tested results are archived. Hallmark enrichment used the signed Wald statistic and fgsea; 37 sets met FDR < 0.05.

The healing analysis used the 13 DFU libraries. The unadjusted model contained healing group, and the sensitivity model additionally contained UM/P2 sample-code family. Both designs were full rank. Neither model included all clinically relevant prognostic covariates. Cook’s distances were compared with the F(0.99; p, m − p) cutoff for each design, where p is the number of model coefficients and m is the sample count. These diagnostics did not trigger automatic sample deletion.

Table B. Individual GSE134431 DFU samples in the exploratory healing analysis.

| **GEO / run** | **Original sample title** | **Healing** | **Code family** |
| --- | --- | --- | --- |
| GSM3946493 SRR9699317 | DFU Patient UM#7W4 | Healer | UM |
| GSM3946494 SRR9699318 | DFU Patient UM#8W4 | Healer | UM |
| GSM3946495 SRR9699319 | DFU Patient UM#15W0 | Healer | UM |
| GSM3946496 SRR9699320 | DFU Patient UM#23W0 | Healer | UM |
| GSM3946497 SRR9699321 | DFU Patient UM#24W0 | Healer | UM |
| GSM3946498 SRR9699322 | DFU Patient UM#30W4 | Healer | UM |
| GSM3946499 SRR9699323 | DFU Patient P2#13 | Healer | P2 |
| GSM3946500 SRR9699324 | DFU Patient UM#27W4 | Nonhealer | UM |
| GSM3946501 SRR9699325 | DFU Patient UM#28W4 | Nonhealer | UM |
| GSM3946502 SRR9699326 | DFU Patient UM#31W4 | Nonhealer | UM |
| GSM3946503 SRR9699327 | DFU Patient P2#01 | Nonhealer | P2 |
| GSM3946504 SRR9699328 | DFU Patient P2#03 | Nonhealer | P2 |
| GSM3946505 SRR9699329 | DFU Patient P2#21 | Nonhealer | P2 |

Original W0/W4 strings are preserved as sample-title information; their clinical timing was not independently reconstructed or added as a verified covariate. Healers include six UM and one P2 sample; nonhealers include three UM and three P2 samples. Code family is imbalanced but not perfectly confounded with healing. In the separate DFU-versus-DFS comparison, code family is structurally confounded with condition.

At FDR < 0.10, the unadjusted model selected 120 genes and the adjusted model 1,471. Their intersection contained 111 genes; nine were unadjusted-only and 1,360 adjusted-only (Jaccard index 0.075). Across 23,399 genes with finite effects in both models, Spearman ρ was 0.755 and direction agreement 78.8%. In the 1,480-gene union of selected sets, ρ was 0.950 and direction agreement 99.7%. Fisher’s exact P for the healing-by-code-family table was 0.266. Cook’s threshold exceedances occurred in 12/13 libraries in the unadjusted model and 11/13 in the adjusted model. These findings indicate distributed influence and sensitivity to estimated precision; they do not establish that adjustment yields a more reliable prognostic signature.

# 5. Microarray analysis and overlap accounting

All 12 GSE80178 arrays were jointly RMA-normalized with oligo. Transcript clusters mapped to one gene symbol were averaged. limma used separate DFU, DFS and nondiabetic-skin coefficients, robust empirical Bayes moderation and a mean–variance trend. Six DFU versus three DFS arrays constituted the primary contrast. GSM2114232 had the largest relative log-expression spread and separated in PCA. Its post hoc exclusion produced Spearman ρ = 0.956 for effect estimates, retained 99.6% of primary FDR-positive genes below FDR 0.05 and preserved their directions.

There were 27,277 microarray symbols and 17,502 shared symbols across the bulk analyses. The original integration code dropped identifiers without symbols and retained the smallest-adjusted-P-value entry for duplicated RNA sequencing symbols. Of 4,704 selected RNA sequencing identifiers, 1,399 lacked a symbol. The remaining 3,305 entries mapped to 3,303 unique symbols; 360 were not in the shared universe, leaving 2,943 for the selected-gene directional comparison. This mapping rule is result-dependent and may affect the compared set.

Direction agreement was 2,065/2,943 (70.2%). A total of 1,254 had concordant directions and nominal microarray P < 0.05; 1,028 also had microarray FDR < 0.05. Within all 17,502 shared symbols, 2,943 met the primary criteria and 5,541 met microarray FDR < 0.05. The Venn intersection comprised 1,210 genes, of which 1,028 were concordant and 182 opposite in direction. RNA sequencing only, microarray only and neither counts were 1,733, 4,331 and 10,228, respectively. Spearman ρ = 0.2875 summarizes all shared effect ranks. The microarray selection has no fold-change cutoff in the main overlap; S2 File also reports cutoffs of 0.5 and 1. Both correlation and thresholded overlap have platform- and power-dependent limitations.

Five Hallmark sets met primary FDR < 0.05, microarray FDR < 0.10 and concordant NES directions: tumor necrosis factor-alpha/nuclear factor-kappa B signaling, hypoxia, glycolysis and inflammatory response were positive; interferon-alpha response was negative. These criteria are asymmetric across cohorts and do not imply equal evidence strength.

# 6. Single-cell patient mapping and replication

The 33 foot matrices represented 26 participants: nine without diabetes, six with diabetes but no DFU, seven healers and four nonhealers. Their 94,325 public filtered cells passed the reapplied ≥200 detected genes and ≤50% mitochondrial-UMI criteria. Genes detected in fewer than three cells were removed. Log-normalized expression, 3,000 highly variable genes, 50 principal components, a 20-neighbor graph, UMAP and Leiden clustering supported broad annotation only. Raw UMIs were retained for inference.

Raw counts were summed by participant and cell type, merging repeated foot specimens. Profiles required ≥50 cells and ≥10,000 UMIs. A cell-type contrast required ≥3 participants per group. edgeR used group-only designs, filterByExpr with minimum count 10, TMM normalization, robust dispersion estimation and quasi-likelihood tests. Gene selection required FDR < 0.05 and absolute log2 fold change ≥1. The eligible sample counts below are the actual replication after these filters.

Table C. Eligible patient counts and gene tests across all cell-type contrasts.

| **Cell type** | **Contrast** | **Reference n** | **Comparison n** | **Genes tested** | **Selected genes** |
| --- | --- | --- | --- | --- | --- |
| Endothelial | DFU vs diabetes without DFU | 5 | 11 | 11,638 | 0 |
| Endothelial | Nonhealer vs healer | 7 | 4 | 11,433 | 0 |
| Fibroblast | DFU vs diabetes without DFU | 6 | 11 | 12,617 | 0 |
| Fibroblast | Nonhealer vs healer | 7 | 4 | 13,934 | 0 |
| Keratinocyte | DFU vs diabetes without DFU | 5 | 10 | 12,193 | 12 |
| Keratinocyte | Nonhealer vs healer | 6 | 4 | 12,343 | 0 |
| Myeloid | DFU vs diabetes without DFU | 3 | 9 | 9,117 | 0 |
| Smooth muscle / pericyte | DFU vs diabetes without DFU | 5 | 11 | 12,779 | 0 |
| Smooth muscle / pericyte | Nonhealer vs healer | 7 | 4 | 12,845 | 0 |
| T / NK | DFU vs diabetes without DFU | 5 | 11 | 5,715 | 0 |
| T / NK | Nonhealer vs healer | 7 | 4 | 8,500 | 0 |

Reference groups are diabetes without DFU or healer; comparison groups are DFU or nonhealer. “Selected” means FDR < 0.05 and absolute log2 fold change ≥1. The other four annotated lineages did not meet the replication requirement. Patient-level group-only models do not remove all clinical confounding.

The 12 selected keratinocyte genes were *CARD6*, *SPRR2A*, *KYNU*, *SERPINB4*, *KCNJ15*, *S100A9*, *CLEC7A*, *C4orf48*, *C2CD4A*, *AKR1B10*, *SERPINB13* and *S100A8* (symbols retained as annotated in the archived data). No other eligible lineage met the individual-gene criteria, and none met the healing-status criteria. These differences in significance are not formal between-lineage effect comparisons. Other lineages exhibited Hallmark-level associations. Fig 8 separately displays the first 20 bulk-supported genes ordered by microarray FDR status and adjusted P values; it shows natural log(1 + mean CPM) in eligible DFU profiles, not the 12-gene keratinocyte test result.

# 7. More specific functional annotation

The revision analyzed the 1,028 stricter concordant genes separately by direction (561 up and 467 down). Reactome Pathways 2024 and Gene Ontology Biological Process 2026 GMT libraries were downloaded through the Enrichr public gene-set endpoint on 6 September 2026. This supplies curated pathway and biological-process annotations; it is not an MSigDB C2/C5 ranked enrichment analysis. The libraries contain 2,105 and 5,954 terms before background filtering. Frozen files and their SHA-256 hashes are included in S2 File.

For each direction and term, N = 17,502 shared symbols; n is the direction-specific query size; K is the number of term genes in the shared universe; and k is the overlap. Terms required 15 ≤ K ≤ 500. The probability is Pr[X ≥ k] for X following a hypergeometric distribution with N, K and n. Fold enrichment is (k/n)/(K/N). Benjamini–Hochberg adjustment jointly covered all 7,764 tests across both directions and both collections, including zero-overlap tests. Overlapping terms are not statistically independent biological mechanisms.

Twenty-six terms met global FDR < 0.05: 18 Gene Ontology and eight Reactome terms, all for upregulated genes. Formation of the cornified envelope and keratinization each overlapped 29 genes (FDR 1.86 × 10⁻¹⁴ and 2.00 × 10⁻¹⁴). Other terms included keratinocyte differentiation, epidermal cell differentiation, granulocyte chemotaxis and regulation of wound healing. S7 Fig displays the five smallest-FDR terms per collection; all tests and membership genes are in S2 File. This post hoc functional description depends on the selected bulk gene set and is not new independent replication or evidence of pathway activation.

# 8. Additional descriptive cohort: GSE199939

The GEO series describes 10 DFU samples and 11 healthy foot-skin controls obtained during comparable foot surgery. Individual sample titles use “diabetic foot skin”; the analysis uses the clinical grouping in the series description and linked publication. The deposited file is GSE199939_gene.tpm.matrix.annot_-_21.txt.gz. Duplicate gene-symbol TPMs were summed, symbols with TPM ≥1 in at least three samples were retained, and the difference in mean log2(TPM + 1) was computed for DW1–DW10 minus N1–N11. TPM was not rounded or passed to DESeq2, and no gene-level inferential P values were reported.

There were 19,752 eligible symbols and 14,892 shared with the main comparison. The stricter supported set contributed 823 represented genes, of which 611 (74.2%) had the same effect direction. The all-shared descriptive rank correlation was 0.370. Different expression scales, healthy rather than diabetic controls, and clinical heterogeneity prohibit interpreting this as a direct DFU-versus-DFS replication. The 205 supported genes absent after mapping/filtering do not enter the 823-gene denominator. The full descriptive output is supplied, rather than only agreeing genes.

# 9. Spatial-panel coverage and limits of mechanistic inference

The original single-cell publication also reported spatial study GSE166120. We downloaded its Alpha CTA v2.0 probe-panel definition and checked candidate symbols against target DisplayName and probe SystematicName entries. Only *S100A8*, *S100A9* and *CLEC7A* of the 12 keratinocyte candidates were represented. The other nine were not listed in the panel. This is an exact-symbol panel-coverage audit, not spatial differential-expression analysis. The source experiment included two healing and two nonhealing patients, with multiple regions from each patient, and no diabetic nonulcerated comparator. Its published fibroblast and macrophage associations provide prior biological context but cannot be presented as new validation of this full gene list.

No new spatial model or ligand–receptor communication model was fitted. Pooling regions as independent patients would overstate replication. Broad cell annotations and expressed ligand/receptor pairs would in any case not establish a causal interaction. Testing mechanisms will require suitable spatial coverage, independent patient replication, refined cell states and functional experiments.

# 10. Reproducibility and source references

The original models and raw-count preparation are documented in the Zenodo deposit, including software sessions for R v4.6.1, Bioconductor v3.23 and Python v3.13.13. Revision computations used Python v3.12.13, NumPy v2.3.5, pandas v2.2.3, SciPy v1.17.0 and Matplotlib v3.10.8. From S2 File, run “python analyze_revision.py --root .” and “python make_revision_figures.py --root .”. The supplied frozen inputs allow these commands to execute without network retrieval. S1 Table remains an unchanged record of the original analyses and should be read with the updated definitions above.

Original single-cell and spatial source study: https://doi.org/10.1038/s41467-021-27801-8

Additional cohort source study: https://doi.org/10.1016/j.intimp.2024.112638

Reactome resource: https://doi.org/10.1093/nar/gkad1025

Gene Ontology resource: https://doi.org/10.1093/genetics/iyad031

Enrichr resource: https://doi.org/10.1002/cpz1.90
